# Life Prediction Model for High-Cycle and Very-High-Cycle Fatigue of Ti-6Al-4V Titanium Alloy Under Symmetrical Loading

**DOI:** 10.3390/ma18143354

**Published:** 2025-07-17

**Authors:** Xi Fu, Lina Zhang, Wenzhao Yang, Zhaoming Yin, Jiakang Zhou, Hongwei Wang

**Affiliations:** 1 School of Machinery and Automation, Weifang University, Weifang 261061, China; 20190005@wfu.edu.cn (X.F.); 20230021@wfu.edu.cn (Z.Y.); 20220087@wfu.edu.cn (J.Z.); 2 Department of Mechanical Engineering, Hebei University of Water Resources and Electric Engineering, Changzhou 061001, China; zhanglina134679@126.com; 3 Weichai Power Co., Ltd., Weifang 261000, China; yangwz@weichai.com

**Keywords:** Ti-6Al-4V titanium alloy, high-cycle fatigue, very-high-cycle fatigue, continuous damage mechanics, fatigue life prediction model, optimization of fatigue model parameters

## Abstract

The Ti-6Al-4V alloy is a typical α + β type titanium alloy and is widely used in the manufacture of aero-engine fans, compressor discs and blades. The working life of modern aero-engine components is usually required to reach more than 10^8^ cycles, which makes the infinite life design based on the traditional fatigue limit unsafe. In this study, through symmetrical loading high-cycle fatigue tests on Ti-6Al-4V titanium alloy, a nonlinear cumulative damage life prediction model was established. Further very-high-cycle fatigue tests of titanium alloys were carried out. The variation law of plastic strain energy in the evolution process of very-high-cycle fatigue damage of titanium alloy materials was described by introducing the internal stress parameter. A prediction model for the very-high-cycle fatigue life of titanium alloys was established, and the sensitivity analysis of model parameters was carried out. The results show that the established high-cycle/very-high-cycle fatigue models can fit the test data well. Moreover, based on the optimized model parameters through sensitivity analysis, the average error of the prediction results has decreased from 59% to 38%. The research aims to provide a model or method for predicting the engineering life of titanium alloys in the high-cycle/very-high-cycle range.

## 1. Introduction

Ti-6Al-4V is a typical α + β type titanium alloy, containing 6% α-stabilizing aluminum and 4% β-stabilizing vanadium. It is widely used in the manufacture of aero-engine fans, compressor disks and blades [1]. During the service life of an aero-engine, the blades of the fan and compressor are prone to generate vibration stress with a high frequency and low load, and the number of cyclic loading cycles is usually above 10^7^. However, most existing studies on engineering practice are still focused on low-cycle and high-cycle fatigue stages, with the number of cycles not exceeding 10^6^ to 10^7^ [2]. This compromises the safety of the infinite life design based on traditional *S*-*N* curves and fatigue limits. Through the “High cycle Fatigue Science and Technology Program,” the United States has incorporated the very-high-cycle performance requirements for titanium alloys into the “Engine Structural Integrity Program” (ENSIP) which stipulates that the minimum high-cycle fatigue life of every engine component must be 10^9^ cycles [3].

*S*-*N* curves can preliminarily determine the fatigue properties of materials and have been widely used in the research on high-cycle fatigue life prediction for materials. According to Sonsino [4], analysis of the fatigue properties of materials using *S*-*N* curves can guide structural design and safety analysis. However, when the number of cycles exceeds 10^7^, the traditional *S-N* curve turns at the fatigue limit, leading to significant calculation errors in life prediction under loading below the fatigue limit. To extend the engineering application range of the *S*-*N* curves, Maddox [5] obtained the *S*-*N* curve of forged steel through fatigue testing, analyzed the impacts of the stress ratio and surface roughness on the fatigue strength of the material, and corrected the *S*-*N* curve based on Goodman’s mean stress. Zhou et al. [6] utilized an ultrasonic fatigue testing device to investigate the very-high-cycle fatigue characteristics of welded structures, with fatigue cycles reaching up to 10^10^. Under ultrasonic harmonic resonance conditions, the transient structural stress method for evaluating the fatigue life of welded joints has been adopted, and the theoretical application scope of the principal *S-N* curve method has been extended to the very-high-cycle area. Wang et al. [7] investigated the high-cycle fatigue failure mechanisms of CrMoV and NiCrMoV dissimilar steel welding joints and found that the fatigue *S*-*N* curves of the joints showed a continuous downward trend, but no fatigue limit plateau was observed during the high-cycle fatigue process. Regarding the engineering life prediction of very-high-cycle fatigue and the application of extending the *S-N* curve to the 10^9^ cycle range, there are no clear conclusions or systematic methods.

**Figure 1:**
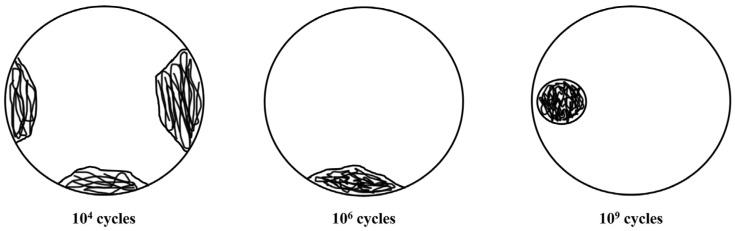
The fatigue crack initiation site under different cycles [31].

In addition, many very-high-cycle performance tests indicate that titanium alloys do not have a fatigue limit in the traditional sense of fatigue. When the cyclic stress amplitude is considerably lower than the yield strength of the material, fatigue fractures still occur after more than 10^7^ cycles, and very-high-cycle fatigue failure cracks are usually initiated inside the specimen [8,9]. Morrissey et al. [10] investigated the very-high-cycle fatigue behavior of titanium alloys under symmetric loading (*R* = −1) and found that the *S*-*N* curves showed a continuous downward trend in both the high-cycle and very-high-cycle ranges. Cleavage facets were not detected in the crack initiation zone, and the initiation mechanism was local slip deformation. Zuo et al. [11] performed fatigue tests, showing that cracks in titanium alloys initiated inside the specimen in both high-cycle and very-high-cycle fatigue ranges. In contrast, under asymmetric loading, very-high-cycle fatigue tests with a positive stress ratio (*R* > 0) showed that cracks also initiated inside the specimen [12], and typical cleavage facets appeared in the crack initiation zone at the fracture. Su et al. [13] investigated the impact of the α-phase volume fraction on the crack initiation location in the Ti-6Al-4V alloy and the variation patterns of very-high-cycle fatigue behavior. Furuya et al. [14] plotted the Goodman diagram for Ti-6Al-4V in the very-high-cycle fatigue range and found that, at a high stress ratio, the fatigue strength was located on the dangerous side of the curve. Yang et al. [15] also found that the degradation of the very-high-cycle fatigue strength was more significant than that of the high-cycle fatigue strength, indicating that higher safety margins must be used for fatigue designs when *R* > −1. Pan et al. [16] reached similar conclusions. In summary, titanium alloys and other metallic materials exhibit significant differences in their high-cycle and very-high-cycle fatigue behaviors. High-cycle fatigue cracks generally initiate on the specimen surface [17,18,19], while very-high-cycle fatigue cracks tend to initiate inside the specimen [20,21,22,23,24,25]. The initiations of fatigue cracks in different life ranges are shown in Figure 1. Wang et al. [26] proposed the point defect precipitation theory to explain the mechanism of crack initiation in the matrix under low stress amplitudes. Since fatigue fracture surfaces are mostly observed under a scanning electron microscope (SEM), this region is generally referred to as the granular bright facet (GBF) region. Research shows that the size of the GBF is related to the fatigue life. The larger the size of the GBF region, the higher the corresponding fatigue life. Therefore, studying the formation mechanism of the GBF region is of great significance for understanding the crack initiation, failure mechanism and life prediction of very-high-cycle fatigue [27,28,29,30]. In the traditional fatigue limit theory, the horizontal asymptote represents the fatigue strength, and when the loading stress is below the horizontal asymptote, the specimen is considered to have infinite fatigue life and does not fail. However, existing high-cycle/very-high-cycle fatigue experiments have shown that metallic materials such as titanium alloys do not exhibit an absolute fatigue limit in the very-high-cycle regime. Currently, there is still a lack of reasonable models or theories to explain the variation laws of high-cycle/very-high-cycle fatigue in titanium alloys, nor has a method been established for engineering fatigue life prediction applicable to such scenarios.

In this study, the evolution of the high-cycle/very-high-cycle fatigue properties and fracture surface analysis of the Ti-6Al-4V titanium alloy were investigated by conducting symmetrical loading fatigue tests. Then, based on the continuum damage mechanics, a nonlinear fatigue damage prediction model was used to fit the high-cycle fatigue test data of the titanium alloy. Subsequently, the very-high-cycle fatigue test was conducted to determine the stress value leading to the plateau zone of high-cycle/very-high-cycle fatigue behavior of the titanium alloy. The evolution of the plastic strain energy during the very-high-cycle fatigue damage process was described by introducing an internal stress parameter, and a very-high-cycle fatigue life prediction model for the titanium alloy was established. Through our research, we aimed to contribute to the existing knowledge of VHCF in titanium alloys and provide a model or method for predicting the engineering life of titanium alloys in the high-cycle/very-high-cycle range, and this model can describe the nonlinear damage accumulation with higher accuracy than the *S-N* curve.

## 2. Experimental Material and Procedures

### 2.1. Materials and Static Tensile Test

**Figure 2:**
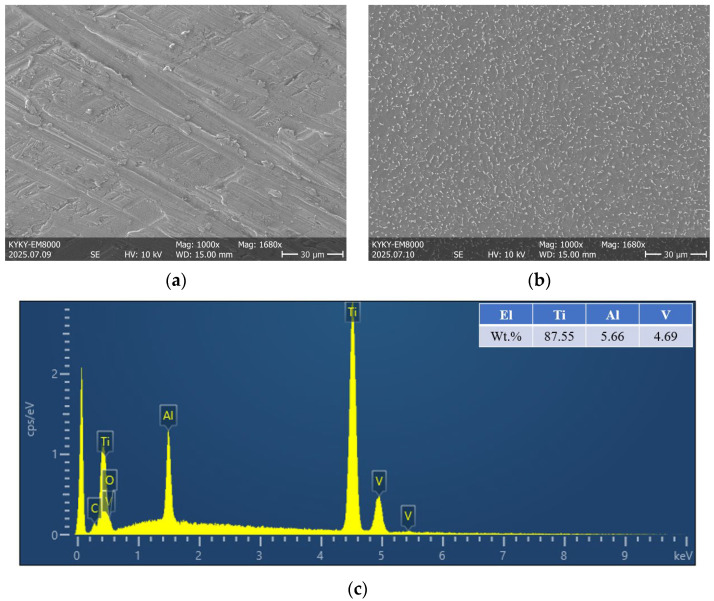
The SEM-EDX microstructure and chemical composition analysis of the Ti-6Al-4V alloy specimen: (**a**) the SEM microstructure of the original surface; (**b**) the SEM microstructure of the corrosive surface; (**c**) the results of EDX analysis of the Ti-6Al-4V alloy specimen surface.

The material investigated in this study is Ti-6Al-4V titanium alloy (Chinese brand TC4, Baoji Wanjia Hongji New Materials Co., Ltd., Baoji, Shanxi, China), whose main chemical composition is shown in Table 1 according to Ref [32], and Figure 2 shows the microstructure and chemical composition analysis of Ti-6Al-4V alloy specimens. The static mechanical property test was conducted to obtain the tensile properties of the Ti-6Al-4V titanium alloy, laying a foundation for the subsequent high-cycle/very-high-cycle fatigue test designs for Ti-6Al-4V.

**Figure 3:**
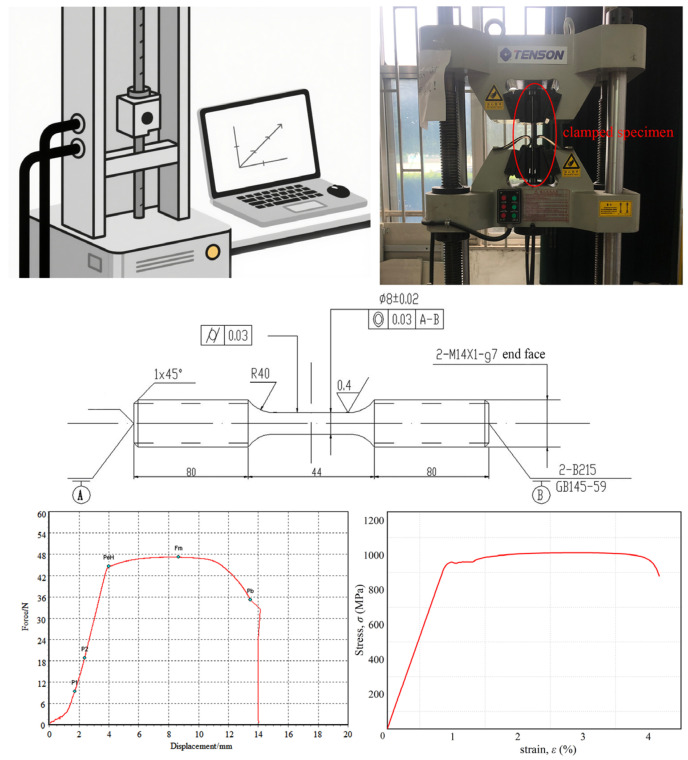
Ti-6Al-4V titanium alloy tensile test system, specimen and results.

The room-temperature tensile specimen was designed according to GB/T 228.1-2010 [33], as shown in Figure 3. The specimen was machined from a φ15 mm rod, the main dimensions of which include an 80 mm long grip section, a 96 mm long gauge section and an R40 mm transition arc connecting the above two parallel sections. The specimen surface was polished with sandpaper before testing. The tensile tests were conducted using an electro-hydraulic servo testing machine (NCS TESTING TECHNOLOGY Co., Ltd., Beijing, China) with a maximum testing force of 600 kN, a maximum static force of 600 kN and a frequency range of 0–100 Hz. The test method involved setting the testing machine to constant displacement control, controlling the strain rate through crosshead displacement and measuring the axial deformation of the specimen. The test was conducted at room temperature, the crosshead displacement rate parameter was 1 mm/min, the sampling frequency of the test data was 10 Hz and the force–displacement curve was recorded during the process. The static tensile properties of the material were obtained, including a strength limit of 999 MPa, a yield strength of 947 MPa and an elastic modulus of 107 GPa.

### 2.2. High-Cycle Fatigue Test and Results

**Figure 4:**
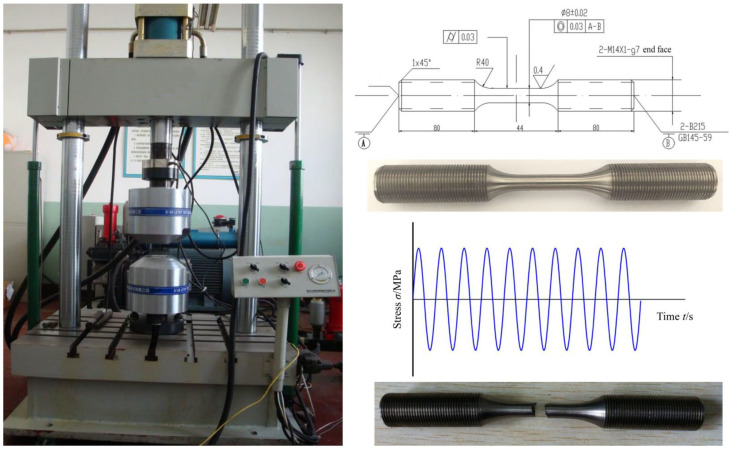
Ti-6Al-4V titanium alloy high-cycle fatigue test systems, specimens and loading methods.

The specimen for titanium alloy high-cycle fatigue tests was machined from Ø20 mm diameter rods. The cross-sectional dimensions of the grip section of the specimen were M14 × 80 mm, and those of the gauge section were Ø8 × 44 mm, with a diameter tolerance requirement of 0.02 mm. A R40 mm transition arc connected the grip and gauge sections. The surface of the gauge section of the machined specimen was polished with sandpaper of grades 240, 600, 1000 and 2000. The high-cycle fatigue tests were conducted using a GPS200 high-frequency fatigue testing machine (Sinotest Equipment Co., Ltd, Changchun, Jilin, China) with the following specifications: a maximum test load of 200 kN, maximum alternating load of 100 kN and loading frequency range of 80–250 Hz. The physical specimen is shown in Figure 4.

The axial tension–compression high-cycle fatigue test was conducted at room temperature with a test frequency of 130 Hz under constant-amplitude loading. The loading method (Figure 4) was symmetrical (stress ratio *R* = −1), with 7 to 9 different stress levels designed based on the single-point method. The maximum stress of the first specimen was set at 0.6 to 0.7 times the tensile strength, and that of the second specimen was about 0.5 to 0.6 times the tensile strength. The stress level was sequentially reduced until the specimen ran out (with >10^7^ cycles, it is the fatigue baseline) at a certain stress level. The test would be halted if the specimen reached 10^7^ cycles without failure. The fatigue-fractured specimen is shown in Figure 4. A total of 12 valid stress-life test datasets were obtained (listed in Table 2).

**Figure 5:**
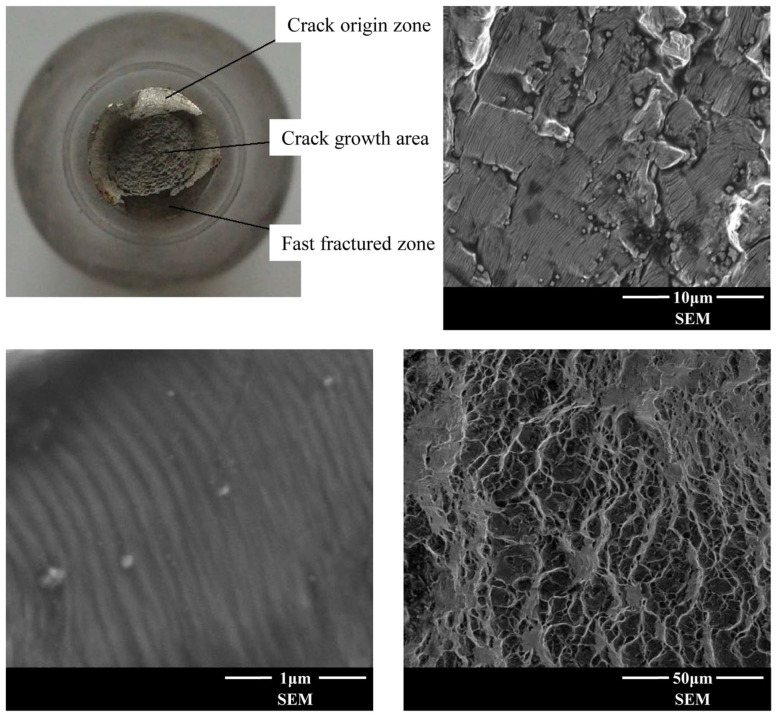
Microscopic characteristics of fracture surface for high-cycle fatigue.

The fracture morphology of the specimen is illustrated in Figure 5, demonstrating three distinct zones in the high-cycle fatigue fracture of the Ti-6Al-4V alloy. The crack initiation site (fatigue origin) first emerged on the specimen surface, propagating inward along the loading direction. This region exhibited a smooth fracture surface, accounting for approximately 25% of the fracture diameter. In the crack propagation zone, the crack advanced perpendicularly to the loading direction at a relatively stable rate, with the fracture surface displaying a radiating pattern that occupied roughly half of the fracture diameter. The final fracture zone, characterized by a quasi-brittle appearance, was positioned opposite to the crack initiation site on the fracture surface.

Microscopic observations of the crack propagation zone in Figure 5 reveal prominent fatigue striations as primary crack features, with some striations deviating from parallel alignment due to lattice structure influences. The instantaneous fracture zone is predominantly composed of dimples, indicating that the failure mechanism of Ti-6Al-4V alloy is dominated by ductile fracture.

### 2.3. Very-High-Cycle Fatigue Test and Results

**Figure 6:**
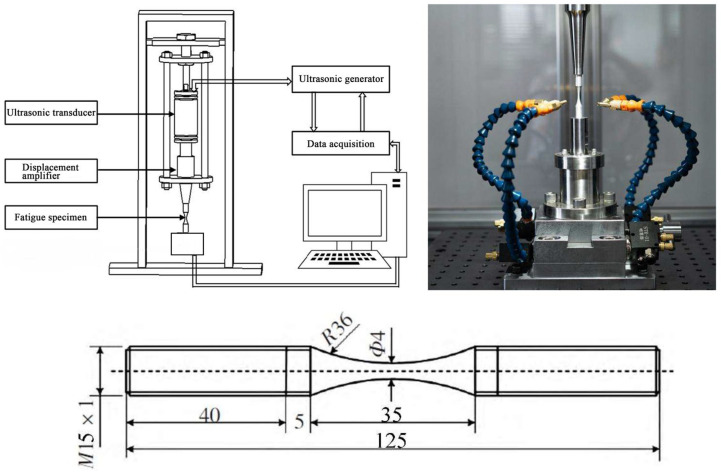
Ti-6Al-4V titanium alloy very-high-cycle fatigue test systems and specimen.

The very-high-cycle fatigue test specimen was designed according to test requirements and machined from a φ15 mm rod. The main dimensions included a 40-mm long grip section, a 35-mm long gauge section and an R36 mm transition arc connecting the above two parallel sections. The specimen surface was polished with sandpaper before testing. The test was conducted using the Shimadzu USF-2000 ultrasonic fatigue test system (Shimadzu Co., Ltd., Tokyo, Japan), which converted electrical signals into weak high-frequency mechanical vibrations (Figure 6). These vibrations were amplified and focused to drive the specimen into resonance, thus shortening the fatigue test cycle. The test frequency was (20 ± 0.5) kHz, and the load–stress ratio was *R* = −1. The test system is shown in Figure 6; a total of 28 valid stress-life datasets were obtained (Table 3).

**Figure 7:**
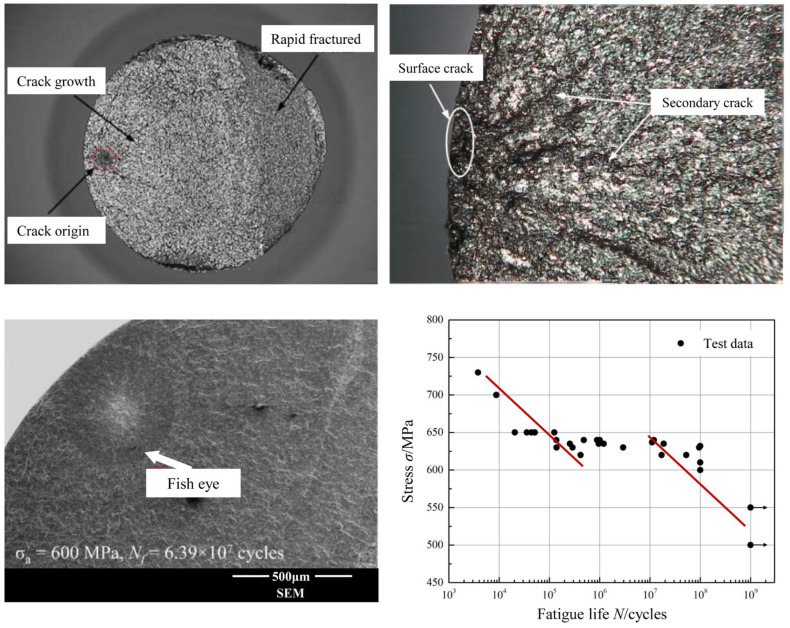
Microscopic characteristics of fracture surface and test results for very-high-cycle fatigue.

Fitting the very-high-cycle fatigue test data using the *S*-*N* curve indicated that the very-high-cycle fatigue performance of the titanium alloy was bilinear, with a plateau zone observed corresponding to the fatigue limit in traditional high-cycle fatigue tests. However, when the load was below the fatigue limit, the specimen did not remain unfractured but fractured after 10^8^ to 10^9^ cycles. Further fracture analysis of the fractured specimen (Figure 7) revealed crack initiation inside the material as well as the presence of uneven and rough internal zones in the fractures. These features caused stress concentration at grain boundaries under the cyclic stress, leading to crack initiation. The cracks propagated under cyclic loading and eventually caused the specimen to fail. When fatigue cracks initiated inside the material, the crack initiation and early propagation zones exhibited the typical “fish-eye” fatigue morphology [31].

## 3. Life Prediction Model of the Ti-6Al-4V Titanium Alloy in the High-Cycle Fatigue Regime

### 3.1. Constitutive Model of Continuous Damage Mechanics

The one-dimensional surface damage theory describes the degree of damage to a material using the bearing area on its surface. Under the assumption of material isotropy, the expression for the one-dimensional damage parameter is as follows:

(1) $$D=\frac{S_{D}}{S}=1-\frac{S_{e}}{S},$$

where *S* is the total cross-sectional area, *S*_D_ is the area occupied by small defects and *S*_e_ is the effective bearing area.

Based on the one-dimensional damage theory, three-dimensional damage analysis describes intermediate stages of the damage evolution by considering its nonlinear nature. According to Lemaitre’s strain equivalence assumption, the effective stress in the damaged state is used to replace the nominal stress in the undamaged state when the constitutive relations with damage are established. The following equation can be deduced [34]:

(2) $$\sigma_{e}=\frac{\sigma }{1-D}.$$

According to the elastoplastic stress–strain relationship, the total strain includes the elastic and plastic strains. The total strain rate can also be decomposed into elastic and plastic strain rates as in [35]:

(3) $${\dot{\varepsilon }}_{ij}={\dot{\varepsilon }}_{ij}^{e}+{\dot{\varepsilon }}_{ij}^{p},$$

where the plastic term can be expressed as follows:

(4) $${\dot{\varepsilon }}_{ij}^{p}=\frac{\partial f\left(\sigma -\Omega \right)}{\partial \sigma }{\bar{\dot{\varepsilon }}}_{ij}^{p},$$

where ${\bar{\dot{\varepsilon }}}_{ij}^{p}$ is the equivalent strain rate and Ω is the total back stress.

Based on the irreversible thermodynamics theory, the entire damage evolution process can be associated with one state variable, which can be described by the state potential. For metallic materials, the state potential per unit mass can be defined using the Helmholtz free energy, expressed as follows:

(5) $$\psi =\psi \left(\varepsilon_{ij},T,r,D\right),$$

where *T* is the temperature, *r* is the internal variable and *D* is the damage parameter.

Further decomposing the above state potential into uncoupled elastic and plastic parts, and considering the damage parameter only in the elastic phase, we have the following:

(6) $$\psi =\psi^{e}\left(\varepsilon_{ij}^{e},T,D\right)+\psi^{p}\left(T,\alpha_{ij},r\right),$$

(7) $$\psi^{e}=\frac{1}{\rho }\left[\frac{1}{2}\alpha_{ijkl}\varepsilon_{ij}^{e}\varepsilon_{kl}^{e}\left(1-D\right)\right],$$

where $\alpha_{ijkl}$ is the fourth-order elastic stiffness tensor and ρ is the density.

The fatigue damage evolution in the material is an irreversible process that satisfies both the first and second laws of thermodynamics. Under isothermal (*T* = 0) or adiabatic (∆*T* = 0) conditions, a local entropy inequality, namely, the Clausius–Duhem inequality, exists for infinitesimal strains:

(8) $$\sigma_{ij}{\dot{\varepsilon }}_{ij}-\rho \dot{\psi }\geq 0.$$

Without considering the impact of temperature on the material, and assuming that the material is isotropic in the elastic phase and the resulting damage is also isotropic, we can transform Equation (5) into the following:

(9) $$\psi =\frac{\partial \psi }{\partial \varepsilon_{ij}}\varepsilon_{ij}+\frac{\partial \psi }{\partial r}\dot{r}+\frac{\partial \psi }{\partial D}\dot{D}.$$

Substituting Equations (3) and (9) into Equation (8), we have the following:

(10) $$\left(\sigma_{ij}-\rho \frac{\partial \psi }{\partial \varepsilon_{ij}^{e}}\right){\dot{\varepsilon }}_{ij}^{e}+\sigma_{ij}{\dot{\varepsilon }}_{ij}^{p}-\frac{\partial \psi }{\partial r}\dot{r}-\frac{\partial \psi }{\partial D}\dot{D}\geq 0.$$

Because the parameters in Equation (10) are independent of each other, the constitutive relation can be further derived as follows:

(11) $$\sigma_{ij}=\rho \frac{\partial \psi }{\partial \varepsilon_{ij}}.$$

According to the Clausius–Duhem inequality, the dissipation function is defined as ϕ:

(12) $$\phi =\sigma_{ij}{\dot{\varepsilon }}_{ij}-\rho \left(\dot{\phi }+s\dot{T}\right)-\frac{q}{T}\cdot \mathrm{grad}T\geq 0,$$

which can be transformed into the following:

(13) $$\dot{\phi }=\frac{\partial \phi }{\partial \varepsilon^{e}}{\dot{\varepsilon }}^{e}+\frac{\partial \phi }{\partial D}\dot{D}+\frac{\partial \phi }{\partial T}\dot{T}.$$

The damage process of the material is irreversible. Using the Legendre-Fenchel transform, the dissipative potential $\phi^{*}$ is further derived as follows:

(14) $$\phi^{*}=\phi^{*}\left(Y,\pi ,D,r\right),$$

where π is the microplastic strain and *r* is the dual variable of π.

$\phi^{*}$ is the residual function of ϕ and can be considered the dissipative residual potential of the material. Within the framework of the generalized standard material theory, according to the normality rule for internal variables, the damage evolution equation for the material is given by the following:

(15) $$\dot{D}=\frac{\partial \phi^{*}}{\partial Y}.$$

### 3.2. High-Cycle Fatigue Model Based on Continuous Damage Theory

In the high-cycle fatigue range, the parameters describing high-cycle fatigue loading (stress amplitude $\sigma_{a}$ and mean stress $\sigma_{m}$) are associated with the damage variable *D* to describe the continuity of the damage parameters during the fatigue process. Its generalized differential expression is defined as follows:

(16) $$\frac{dD}{dN}=f^{-1}\left(\sigma_{a},\sigma_{m},D\right)\cdot g\left(\sigma_{a},\sigma_{m}\right),$$

where *g* describes the relationship between loading parameters and function *f* describes the inseparable relationship between load σ and damage *D*, reflecting the nonlinear accumulation of damage and loading sequence effect. The specific functional expression is as follows:

(17) $$dD={\left[1-{\left(1-D\right)}^{\beta +1}\right]}^{\alpha }{\left[\frac{\sigma_{a}}{M_{0}\left(1-b_{0}\sigma_{m}\right)\left(1-D\right)}\right]}^{\beta }dN,$$

where *β*, *M*_0_ and *b*_0_ are model parameters characterizing the fatigue properties of the material and parameters $\sigma_{a}$ and $\sigma_{m}$ characterizing the load can be calculated according to the following mathematical equations: $\sigma_{a}=\sigma_{\max}\left(1-R\right)\;∕2$, $\sigma_{m}=\sigma_{\max}\left(1+R\right)\;∕2$ and $R=\sigma_{\min}∕\sigma_{\max}$; here, *R* is the stress ratio, $\sigma_{\max}$ is the maximum stress of the alternating load and $\sigma_{\min}$ is the minimum stress of the alternating load. *α* characterizes the cyclic nature of the alternating load, associating the damage with the load:

(18) $$\begin{array}{l} \alpha =1-H\langle \frac{\sigma_{\max}-\sigma_{1}\left(\sigma_{m}\right)}{\sigma_{b}-\sigma_{\max}}\rangle \\ \sigma_{1}\left(\sigma_{m}\right)=\sigma_{m}+\sigma_{-l}\left(1-b_{0}\sigma_{m}\right) \end{array},$$

where the arithmetic meaning of operator < > is as follows: when x≤0, 〈x〉=0; when x>0, 〈x〉=x; $\sigma_{-1}$ is the fatigue limit of the material under symmetric loading (*R* = −1); and *H* is a parameter related to *α*, which can be determined by fitting the fatigue test data. According to the differential Equation (17) of the nonlinear continuum damage accumulation model, the model associates loading parameters with damage variables and describes the nonlinear process of damage accumulation.

Under initial conditions, we assume that no damage occurs in the structure, and the damage variable is defined as *D* = 0. As the damage gradually accumulates and causes the structure to fail, the damage variable is defined as *D* = 1. Integrating Equation (17) from *D* = 0 to *D* = 1 yields the following fatigue life equation, which takes into consideration the nonlinear accumulation of damage:

(19) $$N_{f}=\frac{1}{1-\alpha }\frac{1}{1+\beta }{\left[\frac{M_{0}\left(1-b_{0}\sigma_{m}\right)}{\sigma_{a}}\right]}^{\beta }.$$

### 3.3. Discussion

**Figure 8:**
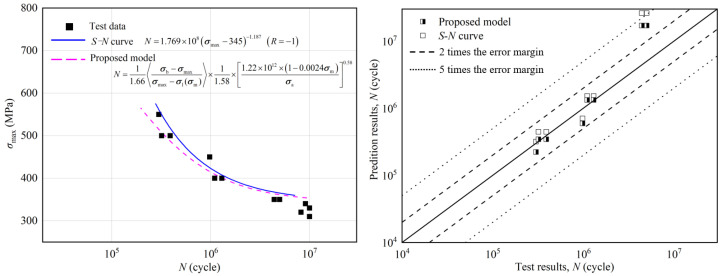
Comparison between proposed model and *S-N* curve of high-cycle fatigue.

Based on the axial tension–compression high-cycle fatigue test of the Ti-6Al-4V titanium alloy, both the *S-N* curve and the high-cycle fatigue prediction model based on the continuum damage theory were used to fit the test data, as shown in Figure 8. The *S-N* curve only characterizes the relationship between stress parameters and life cycles, whereas the nonlinear continuum damage model considers the impacts of mean stress and other loading parameters on fatigue damage accumulation. In the low-to-medium load range (350 to 450 MPa), the predictions from both models are relatively consistent, and their relative errors with the test results are minimal, mostly between the two-fold error bars. However, in the high-load range (450 to 550 MPa), the nonlinear continuum damage model predicts more conservatively, whereas the *S-N* curve prediction is more optimistic, and its relative error is between the five-fold error bars. In addition, when the stress parameter approaches the fatigue limit, the *S-N* curve tends to be a horizontal straight line, indicating that a load below the fatigue limit has no significant impact on damage accumulation. In contrast, the nonlinear damage model presents a smooth curve across the entire load range (500 to 750 MPa). When the load approaches and falls below the fatigue limit, the fatigue properties of the material change continuously with the loading parameters. Therefore, the impact of the applied stress near the fatigue limit on the fatigue damage evolution process of the material was considered.

## 4. Life Prediction Model of Ti-6Al-4V Titanium Alloy in the Very-High-Cycle Fatigue Regime

### 4.1. Very-High-Cycle Fatigue Model Based on Continuous Damage Theory

According to the traditional high-cycle fatigue theory, the fatigue benchmark is set to 10^7^ cycles. If a specimen reaches 10^7^ cycles without failure, then the corresponding load amplitude is considered the fatigue limit. When the load amplitude is below the fatigue limit, no damage occurs, implying an infinite life for the structure. However, according to the results of very-high-cycle fatigue tests on titanium alloys, we found that their fatigue performance can extend to loads below the conventional fatigue limits, and their cycle life can reach 10^8^ to 10^9^ cycles with a plateau zone. In addition, experimental studies show that loads near the fatigue limit have fatigue strengthening and damage deterioration effects. Therefore, the stress amplitude in the plateau zone of the very-high-cycle fatigue test is defined as a low load with strengthening effects. A titanium alloy very-high-cycle fatigue damage prediction model was established to describe more accurately the changes in material behavior in this zone.

Under uniaxial loading, the fatigue life prediction model for nonlinear damage accumulation in the titanium alloy is shown in Equation (19). When the load–stress ratio is *R* = −1, based on the calculation formula for model parameter *α* in the equation, the fatigue life prediction model is simplified as follows:

(20) $$N_{f}=\frac{1}{H}\left(\frac{\sigma_{b}-\sigma_{\max}}{\sigma_{\max}-\sigma_{-1}}\right)\frac{1}{1+\beta }{\left(\frac{2M_{0}}{\sigma_{\max}}\right)}^{\beta }.$$

Because the very-high-cycle fatigue test involves constant-amplitude tension–compression loading with a stress ratio *R* = −1, Equation (20) can be used to fit the very-high-cycle fatigue test data. In the high-load range (where the load exceeds the fatigue limit in traditional high-cycle fatigue tests, corresponding to the left side of the plateau zone in the very-high-cycle fatigue test), parameter $\sigma_{b}$ in Equation (20) is the strength limit of the titanium alloy, and parameter $\sigma_{-1}$ is the load corresponding to the plateau zone in the very-high-cycle fatigue curve (same as the fatigue limit mentioned earlier). In the low-load range (where the load is below the traditional high-cycle fatigue limit, corresponding to the right side of the plateau zone in the very-high-cycle fatigue test), parameter $\sigma_{b}$ in Equation (20) is rewritten as the load amplitude of the plateau zone, denoted as parameter $\sigma_{b}^{*}$; $\sigma_{-1}$ implies that the life can reach the very-high-cycle fatigue limit corresponding to 10^9^ cycles and is denoted as $\sigma_{-1}^{*}$. Then,

(21) $$N_{f}=\frac{1}{H}\left(\frac{\sigma_{b}^{*}-\sigma_{\max}}{\sigma_{\max}-\sigma_{-1}^{*}}\right)\frac{1}{1+\beta }{\left(\frac{2M_{0}}{\sigma_{\max}}\right)}^{\beta }.$$

From a macroscopic perspective of the material, stress–strain is an elastic response in the high-cycle fatigue range. However, in the very-high-cycle fatigue range, if the stress–strain relationship is still considered linear, then we cannot reasonably explain the observed bilinear stress–life relationship in the range of 10^8^ to 10^9^ loading cycles. In addition, microplastic strain may be a key factor affecting the fatigue damage behavior of the material during the very-high-cycle fatigue damage evolution. Therefore, a deterministic relationship is assumed between loading stress and plastic strain in the very-high-cycle fatigue range. Based on the elastoplastic stress–strain relationship, the relationship between the stress amplitude under very-high-cycle fatigue loading and plastic strain is defined as follows:

(22) $$\sigma -\sigma_{-1}=K_{V}{\left(\varepsilon_{p}\right)}^{n_{V}},$$

where *K*_V_ and *n*_V_ are material parameters.

For one loading cycle, the plastic strain energy is expressed as in [36]:

(23) $$W_{P}=\int \left(\sigma -\sigma_{-1}\right)\;d\varepsilon_{P}=\frac{K_{V}}{n_{V}+1}\varepsilon_{P}^{n+1}.$$

As mentioned earlier, during the evolution of the very-high-cycle fatigue behavior, the material exhibits a small plastic strain that can alter the interatomic elastic potential, thus generating a small internal stress $\Delta \sigma^{*}$. Based on the self-equilibrium of the internal stress $\Delta \sigma^{*}$ at the cracked section, we obtain the following:

(24) $$\Delta \sigma^{*}=\alpha_{V}{\left(\frac{x}{A}\right)}^{\beta_{V}},$$

where *x* and *A* are the parameters characterizing the cracked section and *α*_V_ and *β*_V_ are material parameters.

Because the plastic strain energy equals the elastic strain energy corresponding to the maximum internal stress $\Delta \sigma^{*}$ at the cracked section, the corresponding relationship can be expressed as follows:

(25) $$W_{P}=\int_{2A}\frac{{\left(\Delta \sigma^{*}\right)}^{2}}{2E}dA=\int_{-A}^{A}\frac{\alpha_{V}^{2}{\left(\frac{x}{A}\right)}^{2\beta_{V}}}{2E}dx=\frac{A\alpha_{V}^{2}}{E\left(2\beta_{V}+1\right)}.$$

Therefore, we have the following:

(26) $$\Delta \sigma_{\max}^{*}=\alpha_{V}=\sqrt{2E\left(2\beta_{V}+1\right)W_{P}}.$$

Material defects are inevitable, and thus, stress concentration often occurs at defect sites under cyclic loading, leading to an uneven plastic deformation. In addition, the dislocation movement caused by the plastic deformation exacerbates stress concentration. From a microscopic perspective, cyclic loading alters the interatomic elastic potential. By contrast, from a macroscopic view, this can be interpreted as the intrinsic fracture stress of the material reached after superposition of the internal stress $\Delta \sigma^{*}$ with the effective stress $\sigma_{e}$, leading to microcracking. As cyclic loading continues, the microcracks grow, forming a macrocrack with an effective area of ΔA. The internal stress $\Delta \sigma^{*}$ is released, and the equivalent stress $\sigma_{e}$ generated by the external cyclic loading increases. As the internal stress continues to decrease, Equation (26) is transformed into

(27) $$\Delta \sigma_{\max}^{*}=\alpha_{V}\left(\frac{A-0.5\Delta A}{A}\right)=\alpha_{V}\left(1-D\right).$$

Substituting the equation for the equivalent stress, i.e., Equation (2), into Equation (27), we obtain the relationship between the internal stress, effective stress and nominal stress:

(28) $$\Delta \sigma_{\max}^{*}=\alpha_{V}\frac{\sigma }{\sigma_{e}}.$$

Introducing the material parameter α_V_ into the low-load very-high-cycle fatigue life prediction model, we can transform Equation (21) into the following:

(29) $$N_{f}=\frac{1}{H^{\alpha_{V}}}\left(\frac{\sigma_{b}^{*}-\sigma_{\max}}{\sigma_{\max}-\sigma_{-1}^{*}}\right)\frac{1}{1+\beta }{\left(\frac{2M_{0}}{\sigma_{\max}}\right)}^{\frac{\beta }{\alpha_{V}}}.$$

Based on the original fatigue life prediction model (Equation (21)), Equation (29) not only describes the changes in stress–life behavior on the right side of the plateau zone, but also characterizes the impact of the internal stress on the very-high-cycle fatigue damage. Combining Equations (21) and (29), the bilinear very-high-cycle fatigue life prediction model is established as follows:

(30) $$\left\{\begin{array}{l} N_{f}=\frac{1}{H}\left(\frac{\sigma_{b}-\sigma_{\max}}{\sigma_{\max}-\sigma_{-1}}\right)\frac{1}{1+\beta }{\left(\frac{2M_{0}}{\sigma_{\max}}\right)}^{\beta }\;\sigma_{\max}>\sigma_{-1}(\mathrm{high}\;\mathrm{loading})\\ N_{f}=\frac{1}{H^{\alpha_{V}}}\left(\frac{\sigma_{b}^{*}-\sigma_{\max}}{\sigma_{\max}-\sigma_{-1}^{*}}\right)\frac{1}{1+\beta }{\left(\frac{2M_{0}}{\sigma_{\max}}\right)}^{\frac{\beta }{\alpha_{V}}}\;\sigma_{\max}\leq \sigma_{-1}(\mathrm{low}\;\mathrm{loading}) \end{array}\right..$$

Equation (30) extends the nonlinear continuum damage fatigue model from the high-cycle fatigue range to the very-high-cycle fatigue range and takes into account the fatigue damage evolution under low-load conditions.

### 4.2. Parameter Optimization of the Fatigue Model Based on Sensitivity Analysis

In the theoretical research of fatigue life prediction and damage assessment, the prediction model contains model parameters that describe the fatigue characteristics of materials. Some model parameters are relatively sensitive to the prediction results of the fatigue damage model for fitting the parameters; that is, a slight change in the parameter value will cause obvious deviations in the prediction results. Therefore, further sensitivity analysis and optimization of the parameters of the established very-high-cycle fatigue model can greatly improve the accuracy of the predicted life.

Firstly, we used the fatigue test data to fit the parameter values and define the reference value for the sensitivity analysis. The mechanical performance and very-high-cycle fatigue testing results of the titanium alloy indicate that $\sigma_{b}$ has a value of 999 MPa, $\sigma_{-1}$ and $\sigma_{b}^{*}$ are approximately equal to 625 MPa and $\sigma_{-1}^{*}$ has a value of 550 MPa. Other parameters of the model are listed as follows: *H* = 2.23, *β =* 0.4, *M*_0_ = 3.12 × 10^11^, *α*_V_ = 0.42.

Then, the relationship between each model parameter in the fatigue model and the prediction performance was analyzed. Based on the principle of the local sensitivity analysis method and without considering the interactions between various parameters, the influence of the values of model parameters on the prediction results was discussed, and the factors that have a significant impact on the prediction accuracy of the model were determined.

**Figure 9:**
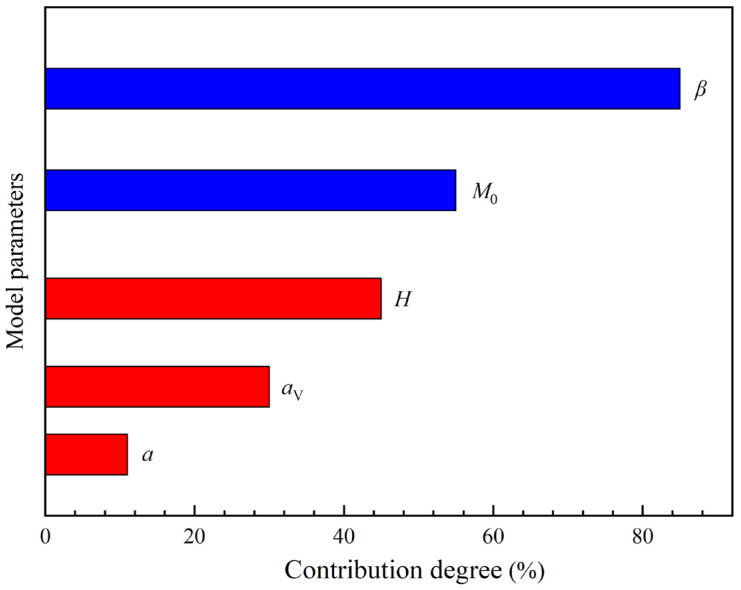
Pareto plot for response of model accuracy.

Figure 9 shows the Pareto graph of the influence of model parameters on prediction accuracy. The horizontal axis represents the contribution of model parameters to prediction accuracy, where blue indicates a positive influence and red indicates a negative influence. In Figure 9, it is found that the parameter *β* has the most obvious impact on the prediction accuracy of the model; this is the exponential parameter of the stress term, characterizing the nonlinear relationship between the stress parameter and the fatigue life in the very-high-cycle fatigue problem. As an exponential parameter, even a slight change of *β* may cause a sudden increase or decrease in the prediction result. Therefore, when fitting the model parameters, the fitting accuracy of the parameters *β* should be considered firstly.

In order to further analyze the influence of material fitting parameters in the fatigue damage model on the prediction accuracy and the mutual influence among the parameters, the Optimal Latin Hypercube Design (Opt LHD) was used to conduct the global sensitivity analysis of the material parameters.

**Figure 10:**
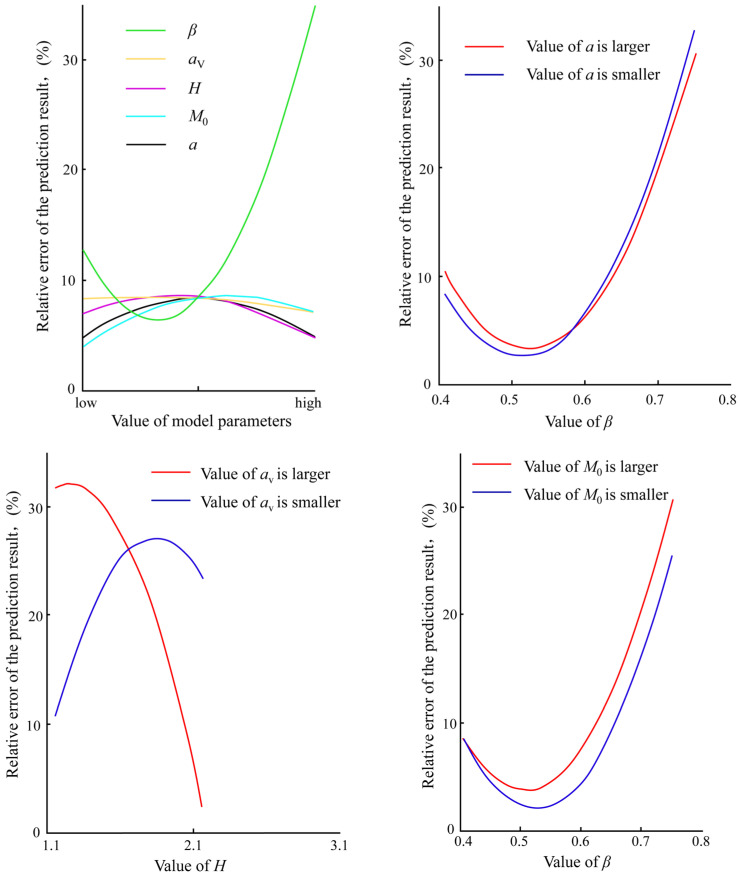
Sensitivity analysis of fatigue parameters.

As shown in Figure 10, it can be found that as the parameter *β* changes from small to large, the prediction accuracy of the model first decreases and then increases. Parameters *H*, *M*_0_ and *α*_V_ have a certain contribution degree. The variation laws of the main effects of parameters *M*_0_ and α are relatively similar. As the parameter values gradually increase, the model prediction error first increases and then decreases. When the parameter values are small, the calculation accuracy of the model is relatively high. Conversely, the variation law of parameter *H* is opposite to that of *M*_0_ and *α*. Although the model accuracy also decreases first and then increases with the increase in parameter values, when the value of *H* is large, it enables the model to have a lower calculation error. Additionally, the parameter α_V_ is the one that contributes the least to the prediction accuracy of the model and has no significant impact on the final calculation result. An interaction effect analysis of fatigue characteristic parameters was conducted as well. The results show that there is obvious interaction between the parameters *H*, *β*, *M*_0_ and *α*_V_. The variation law of the model prediction accuracy with fatigue characteristic parameters is rather complex. Through the local and global sensitivity analysis of the model parameters, it can be found that the values of the model fitting parameters play an important role in the influence on the prediction accuracy of the model. Based on the comprehensive consideration of the constraint conditions of the parameters and reliable test data, an appropriate optimization analysis method was adopted to determine the values of the model parameters, which improves the accuracy of the prediction results to a certain extent and makes it applicable to the fatigue damage analysis of engineering structures.

**Figure 11:**
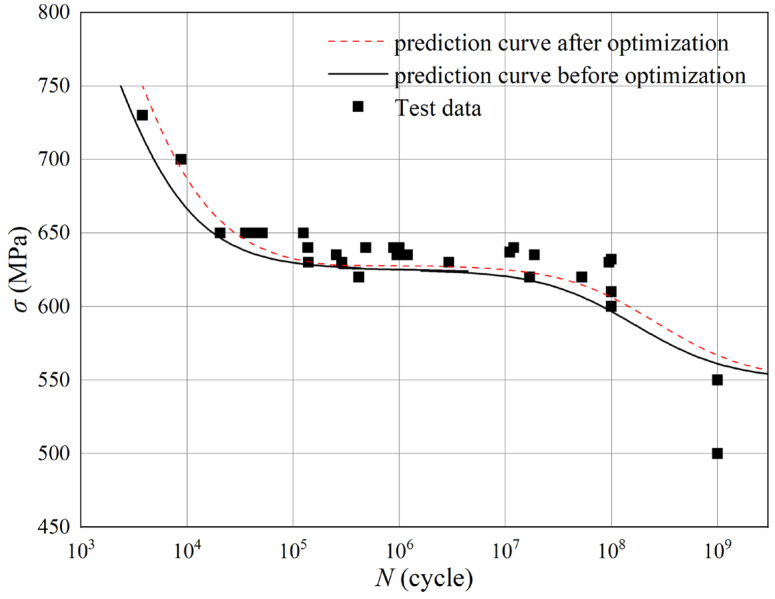
Comparison between optimized and original model curve of very-high-cycle fatigue.

In this study, we aimed to improve the prediction accuracy of the fatigue model by optimizing the fitted material parameters in the model. Therefore, in the optimization process, the optimization objective was determined to minimize the relative error between the model predictions and experimental values. Based on the parameter sensitivity analysis of the model, the baseline values of each parameter in the optimization calculation were defined. For parameters *H*, *β*, *M*_0_ and *α*_V_, which are more sensitive to the model prediction accuracy, the high and low levels of each factor in the optimization analysis were set as ±30% of the baseline values, respectively. Considering that this research belongs to single-objective optimization and the number of optimization parameters is limited, the Nonlinear Programming by Quadratic Lagrangian (NLPQL) algorithm was selected as the solver of the optimization algorithm, which has high stability. Its core algorithm is the quadratic programming method of the SQP sequence. The basic idea was to expand the objective function as a second-order Taylor series. It was assumed that the objective function and the constraint conditions are continuously differentiable and the constraint conditions are linearized. The nonlinear problem was transformed into a quadratic programming problem. The next design point was obtained by solving the quadratic programming problem, and then a linear search was conducted on the two alternative optimization functions. The optimization values of each parameter are listed in Table 4 and the fitting curves of Formula (30) before and after optimization are shown in Figure 11. The prediction accuracy was significantly enhanced, with the average error of the calculated prediction results decreasing from 59% to 38%.

## 5. Conclusions

In this study, a static mechanical tensile test and high-cycle/very-high-cycle fatigue tests under symmetrical loading were carried out on the Ti-6Al-4V titanium alloy. The variation laws of the high-cycle/very-high-cycle fatigue characteristics of the titanium alloy were analyzed and the fracture surfaces were observed. Then, based on the continuum damage mechanics, a high-cycle/very-high-cycle fatigue damage prediction model for the titanium alloy was established, and a sensitivity analysis and optimization of the model parameters were conducted. The main conclusions are as follows:

1. Through the microscopic observation of the high-cycle fatigue crack propagation zone of the titanium alloy, obvious fatigue striations can be observed. Due to the crystal lattice, some fatigue striations are not parallel. High-cycle fatigue cracks initiate at several adjacent positions on the surface of the specimen. For the fracture morphology of very-high-cycle fatigue, the fatigue cracks all initiate inside the specimen, and typical “fish-eye” fracture morphology characteristics are formed.
2. The high-cycle fatigue model based on nonlinear continuum damage mechanics is a smooth curve within the entire load range (500 MPa to 750 MPa) of the test. When the applied load is close to the fatigue limit, the fatigue characteristics of the material change continuously with the loading parameters. At the same time, the influence of the applied stress on the fatigue damage development process of the material is considered, and the prediction accuracy is higher than that of the *S-N* curve.
3. For the very-high-cycle fatigue model based on nonlinear continuum damage mechanics, by introducing the internal stress parameter to describe the variation law of the plastic strain energy during the very-high-cycle fatigue damage evolution process of the titanium alloy material, the law of damage accumulation and development of high-cycle/very-high-cycle fatigue of the titanium alloy is revealed. In addition, the prediction accuracy of the model after parameter optimization is significantly improved, and the average error of its prediction results is reduced from 59% to 38%.

## Figures and Tables

**Table 1:** The chemical composition of the Ti-6Al-4V titanium alloy [32].

Chemical Composition (Weight Percent)								
Main Constituents/%			Impurity (No More Than)/%					
Ti	Al	V	Fe	C	H	O	N	Other Elements
Residual	5.5~6.75	3.5~4.5	0.30	0.08	0.015	0.2	0.05	0.40

**Table 2:** The results of the high-cycle fatigue test for the Ti-6Al-4V titanium alloy (*R* = −1).

No.	Stress /MPa	Cycles	State	No.	Stress /MPa	Cycles	State
1	550	3.00 × 10^5^	fractured	7	350	4.40 × 10^6^	fractured
2	500	3.20 × 10^5^	fractured	8	350	5.00 × 10^6^	fractured
3	500	3.90 × 10^5^	fractured	9	340	9.10 × 10^6^	fractured
4	450	9.80 × 10^5^	fractured	10	330	1.00 × 10^7^	Un-fractured
5	400	1.10 × 10^6^	fractured	11	320	8.20 × 10^6^	fractured
6	400	1.30 × 10^6^	fractured	12	310	1.00 × 10^7^	Un-fractured

**Table 3:** The results of the very-high-cycle fatigue test for the Ti-6Al-4V titanium alloy (*R* = −1).

No.	Stress /MPa	Cycles	State	No.	Stress /MPa	Cycles	State
1	730	3.85 × 10^3^	fractured	15	640	4.84 × 10^5^	fractured
2	700	8.79 × 10^3^	fractured	16	630	2.94 × 10^6^	fractured
3	650	2.05 × 10^4^	fractured	17	635	1.88 × 10^7^	fractured
4	650	4.40 × 10^4^	fractured	18	640	1.38 × 10^5^	fractured
5	650	3.57 × 10^4^	fractured	19	620	5.26 × 10^7^	fractured
6	650	1.25 × 10^5^	fractured	20	640	8.84 × 10^5^	fractured
7	630	2.88 × 10^5^	fractured	21	635	9.56 × 10^5^	fractured
8	600	1.20 × 10^8^	Un-fractured	22	635	2.55 × 10^5^	fractured
9	630	1.39 × 10^5^	fractured	23	632	1.06 × 10^8^	Un-fractured
10	610	1.20 × 10^8^	Un-fractured	24	635	1.20 × 10^6^	fractured
11	620	4.18 × 10^5^	fractured	25	640	1.21 × 10^7^	fractured
12	620	1.70 × 10^7^	fractured	26	640	1.03 × 10^6^	fractured
13	630	9.50 × 10^7^	fractured	27	550	1.00 × 10^9^	Un-fractured
14	650	5.14 × 10^4^	fractured	28	500	1.00 × 10^9^	Un-fractured

**Table 4:** Results of optimized parameters.

Model Parameters	Results Before Optimization	Results After Optimization
*β*	0.40	0.42
*H*	2.23	1.88
*M*_0_	3.12 × 10^11^	4.08 × 10^11^
*α*_V_	0.43	0.44

## Data Availability

The original contributions presented in this study are included in the article. Further inquiries can be directed to the corresponding author.
